# Towards accurate detection and genotyping of expressed variants from whole transcriptome sequencing data

**DOI:** 10.1186/1471-2164-13-S2-S6

**Published:** 2012-04-12

**Authors:** Jorge Duitama, Pramod K Srivastava, Ion I Măndoiu

**Affiliations:** 1 Department of Computer Science & Engineering, University of Connecticut, 371 Fairfield Rd, Unit 2155, Storrs, CT, 06269-2155, USA; 2 Centre of Microbial and Plant Genetics, Katholieke Universiteit Leuven, Gaston Geenslaan 1 - Box 2471, 3001 Heverlee, Belgium; 3 Department of Immunology and the Center for Immunotherapy of Cancer and Infectious Diseases, University of Connecticut Health Center, 263 Farmington Avenue, Farmington, CT 06030-1601, USA

## Abstract

**Background:**

Massively parallel transcriptome sequencing (RNA-Seq) is becoming the method of choice for studying functional effects of genetic variability and establishing causal relationships between genetic variants and disease. However, RNA-Seq poses new technical and computational challenges compared to genome sequencing. In particular, mapping transcriptome reads onto the genome is more challenging than mapping genomic reads due to splicing. Furthermore, detection and genotyping of single nucleotide variants (SNVs) requires statistical models that are robust to variability in read coverage due to unequal transcript expression levels.

**Results:**

In this paper we present a strategy to more reliably map transcriptome reads by taking advantage of the availability of both the genome reference sequence and transcript databases such as CCDS. We also present a novel Bayesian model for SNV discovery and genotyping based on quality scores.

**Conclusions:**

Experimental results on RNA-Seq data generated from blood cell tissue of three Hapmap individuals show that our methods yield increased accuracy compared to several widely used methods. The open source code implementing our methods, released under the GNU General Public License, is available at http://dna.engr.uconn.edu/software/NGSTools/.

## Background

Recent advances in sequencing technologies have enabled the completion of a growing number of individual genomes, including several cancer genomes (see [1] for a recent review). While whole-genome sequencing provides a near-complete catalog of variants and individual genotypes, sequencing of mRNA transcripts (RNA-Seq) is becoming the method of choice for studying functional implications of genetic variability [2-8]. In particular, RNA sequencing is an important source of information for studying the effect of genetic variation on transcription regulation and establishing causal relationships between mutations and disease. For cancer research, comparison of RNA-Seq data generated from normal tissue and tumor samples can provide the information needed to discover driver mutations or to find new therapy targets [9].

Analysis of RNA-Seq data poses several challenging computational problems [10]. First, eukaryotic mRNA transcripts are typically the result of splicing, whereby non-coding regions called introns are removed from the pre-mRNA molecule. This makes the use of tools for mapping of DNA reads to the reference genome like Maq [11] or Bowtie [12] not suitable for finding the genomic location of reads spanning splicing sites. Several methods based on spliced alignment have been proposed to identify splicing sites and assemble full transcripts [7,13-17], however these methods incur a high computational cost and require very high sequencing depth, typically with paired reads. Even when accurate read mapping is achieved, differences in transcription levels result in unequal sequencing depths of different transcripts, making it difficult to identify variants in regions transcribed at low levels. Although it is possible to overcome this difficulty by sequencing both genomic DNA and mRNA and identifying variants from the genomic DNA reads using standard methods, when the interest is in expressed variants it is significantly more cost effective to identify them directly from mRNA reads [18].

Our main contributions are an efficient strategy for accurate mapping of mRNA reads and a new method for single nucleotide variant (SNV) detection and genotyping. Note that we use the term SNV instead of the better known term SNP (Single Nucleotide Polymorphism) because SNPs are normally defined relative to a population and imply a minimum minor allele frequency whereas we are interested in finding and genotyping in an individual all sequence variants that do not match the reference genome sequence, regardless of their frequency in the population. To improve the success rate and accuracy of read mapping, we map mRNA reads against both the reference genome and a transcript library such as the consensus coding sequences (CCDS) database [19] and then combine mapping results using a simple rule set. Our method for SNV detection and genotyping is based on computing, for each locus, conditional probabilities for each of the ten possible genotypes given the reads, and then choosing the genotype with highest posterior probability using Bayes' rule. The underlying probabilistic model assumes independence among reads and fully exploits the information provided by base quality scores. Unlike other widely used Bayesian methods [11,20], we consider all four possible alleles at each position, and do not apply a separate test of heterozygosity.

We validated our methods on publicly available Illumina RNA-Seq datasets generated from blood cell tissue of three individuals extensively genotyped as part of the international Hapmap project [21]. The results indicate that the combined mapping strategy yields improved genotype calling accuracy compared to performing genome or CCDS mapping alone and that our SNV detection and genotyping method is more sensitive than existing methods at equal levels of specificity. We also assess the effect on sensitivity and specificity of commonly used data curation strategies such as read trimming, filtering of read copies to correct for variable transcription levels and PCR artifacts, and imposing minimum allele coverage thresholds.

## Methods

### Mapping strategy for mRNA reads

Mapping mRNA reads against the reference genome using standard mapping programs such as Bowtie [12] or Maq [11] does not require gene annotations but leaves reads spanning exon junctions unmapped. Spliced alignment methods such as [13] could theoretically overcome this difficulty but in practice they are computationally intensive and not well suited for very short reads. On the other hand, mapping against a reference transcript library like the Consensus Coding Sequences Database (CCDS) [19] recovers reads spanning known splicing junctions but fails to recover reads coming from unannotated genes.

We decided to map reads both against the reference genome and the reference transcript library and to implement a custom rule set for merging the two resulting datasets. We implemented two approaches that we called hard merging and soft merging. For hard merging, we require unique alignments against both references and agreement between them while in soft merging we relaxed the uniqueness constraint by requiring a unique alignment to at least one reference and keeping that alignment. For both approaches we keep reads that map uniquely to one reference and do not map to the other one. Table 1 summarizes the decision rules applied to each read by each approach, depending on how the read mapped on each reference and on the concordance between the two alignments. One important issue is how to deal with reads aligned to genes with multiple isoforms. After mapping onto the reference transcriptome, multiple alignments can be reported for some reads not because there exist different genomic locations where the read could come from but because the same genomic location is shared by several different transcripts. After mapping against the transcripts database, our module transfers each alignment to absolute genomic coordinates, splicing accordingly if the alignment spans multiple exons, and then checks for each read with multiple alignments if all of them fall into the same genomic location. If that is the case, just one alignment is kept as unique.

**Table 1:** Decision table for merging of read alignments

Genome mapping	CCDS mapping	Agree?	Hard merge	Soft merge
Unique	Unique	Yes	Keep	Keep
Unique	Unique	No	Throw	Throw
Unique	Multiple	No	Throw	Keep
Unique	Not mapped	No	Keep	Keep
Multiple	Unique	No	Throw	Keep
Multiple	Multiple	No	Throw	Throw
Multiple	Not mapped	No	Throw	Throw
Not mapped	Unique	No	Keep	Keep
Not mapped	Multiple	No	Throw	Throw
Not mapped	Not mapped	No	Throw	Throw

### SNV detection and genotyping

Our new Bayesian method, named *SNVQ*, computes for each locus the posterior probability of each of the ten possible genotypes given the reads. For a locus *i *we let *R_i _*denote the set of mapped reads spanning this locus. In all Bayesian methods, the posterior probability of a genotype is calculated from its prior and conditional probabilities by using the Bayes rule, $P\left(G_{i}|R_{i}\right)=\frac{P\left(R_{i}|G_{i}\right)P\left(G_{i}\right)}{P\left(R_{i}\right)}$. The main difference between models lies in the way conditional probabilities are calculated [22]. Both Maq and SOAPsnp use a different model to calculate probabilities of homozygous and heterozygous genotypes. Maq uses a binomial distribution on the alleles having the two highest counts while SOAPsnp uses a rank test to determine heterozygosity. SOAPsnp also assumes as prior information that the homozygous reference genotype is the most likely one and calculates conditional probabilities based on Illumina specific knowledge about the reads [20]. We decided instead to use a uniform set of assumptions for calculating conditional probabilities of all genotypes. Assuming independence between reads, the conditional probability of genotype *G_i _*can be expressed as a product of read contributions, i.e., $P\left(R_{i}|G_{i}\right)=\prod_{r\in R_{i}}P\left(r|G_{i}\right)$. For a mapped read *r *∈ *R_i _*let *r*(*i*) be the base spanning locus *i *and *ε_r(i) _*be the probability of error sequencing the base *r*(*i*), which we estimated from the quality score *q*(*i*) calculated during primary analysis using the Phred formula *ε_r(i) = _*10^-*q*(*i*)/10 ^[23]. We discarded allele calls with quality scores zero and one. Let *H_i _*and $H_{i}^{\prime }$ be the two real alleles at locus *i*, or in other words, let $G_{i}=H_{i}H_{i}^{\prime }$. The observed base *r*(*i*) could be read from either *H_i _*or $H_{i}^{\prime }$. If there is an error in this read, we assume that the error can produce any of the other three possible bases with the same probability. Thus, the probability of observing a base *r*(*i*) given than the real base is different is *ε_r(i)_*/3 while the probability of observing *r*(*i*) without error is 1 - *ε_r(i)_*.

If *G_i _*is a heterozygous genotype (i.e., $H_{i}\neq H_{i}^{\prime }$) and the observed allele *r*(*i*) is equal to $H_{i}\left(H_{i}^{\prime }\right)$ this outcome could be due to two possible events. Either *r*(*i*) was sampled without error from the haplotype containing $H_{i}\;\left(H_{i}^{\prime }\right)$ or *r*(*i*) was sampled from the haplotype containing $H_{i}^{\prime }\left(H_{i}^{}\right)$ but an error turned it to be equal to *H_i _*(respectively $H_{i}^{\prime }$). Assuming that both haplotypes are sampled with equal probability, the first event happens with probability (1 - *ε_r(i)_*)/2 while the second happens with probability *ε_r(i)_*/6. Using the fact that for homozygous genotypes the probability of observing each possible base does not depend on the haplotype from which the reads are sampled, we obtain the following formula for computing the probability of observing read *r *for each possible genotype:

$$P\left(r|G_{i}=H_{i}H_{i}^{\prime }\right)=\left\{\begin{array}{ccc} 1-\varepsilon_{r\left(i\right)} & , & \text{if}\;H_{i}={H^{\prime }}_{i}=r\left(i\right)\\ \frac{\varepsilon_{r\left(i\right)}}{3} & , & \text{if}\;H_{i}\neq r\left(i\right)\\ & & \wedge \;{H^{\prime }}_{i}\neq r\left(i\right)\\ \frac{1}{2}-\frac{\varepsilon_{r\left(i\right)}}{3} & , & \text{otherwise} \end{array}\right)$$

Note that no matter which is the genotype *G_i_*, the sum of the probabilities *P*(*r*|*G_i_*) over the four possible values of *r_i _*is equal to one. We complete the model by setting prior probabilities based on the expected heterozygosity rate *h *as follows (in all our experiments, we assumed a heterozygosity rate *h *= 0.001):

$$P\left(G_{i}=H_{i}H_{i}^{\prime }\right)=\left\{\begin{array}{ccc} \frac{1-h}{4} & , & \text{if}\;H_{i}={H^{\prime }}_{i}\\ \frac{h}{6} & , & \text{otherwise} \end{array}\right)$$

Finally, a variant is called if the genotype with highest posterior probability is different than homozygous reference. In the next section we show a comparison of results among these methods by reanalyzing a publicly available dataset.

### Software and performance issues

We implemented mapped read merging strategies and SNVQ in Java 1.6 and we packed both programs with a few additional utilities in a single jar file. The open source code, released under the GNU General Public License, is available at http://dna.engr.uconn.edu/software/NGSTools/.

In order to enable integration with other analysis tools we use the SAM format [24] for both the input and the output of mapped read merging. We also sort alignments by chromosome and absolute position to enable efficient processing in subsequent modules and fast merging of results from different lanes if available. SAM files produced by the merging module can be used directly as input for the SAMtools package [24] to produce run statistics, pileup information, and for variants detection. We recommend to run the merging process lane by lane because it needs to load all unique alignments in memory in order to sort them at the end of the process. We used space efficient data structures that allow us to process more than ten million reads in a few minutes, using up to 16 Gb of memory. The code implementing SNVQ is able to receive as input either alignments in SAM format or pileup information in the format described in the SAMtools package. The pileup format is recommended because it enables faster processing and reduces the memory requirements. Our experiments indicate that SNVQ is able to process a whole transcriptome pileup file in about 20 minutes using a single processor and up to 4 Gb of memory.

## Results and discussion

### Experimental setup

We tested the performance of the combined mapping strategies and SNV detection methods on three publicly available Illumina RNA-Seq datasets generated from lymphoblastoid cell lines derived from three individuals extensively genotyped as part of the international Hapmap project [21]: a female with northern and western European ancestry (NA12878, SRA accession numbers SRX000565 and SRX000566), a Yoruban male (NA18498, SRA accession numbers SRX014541, SRX014601, SRX014618, and SRX014653), and a Yoruban female (NA18517, SRA accession numbers SRX014577, SRX014617, SRX014645, and SRX014646). Sequencing and read mapping statistics (using the hard merging strategy) for the three datasets are provided in Table 2.

**Table 2:** Sequencing and read mapping (using hard merge) statistics for the three RNA-Seq datasets used in our experiments

Sample Id	# Lanes	Raw reads	Read length	Initial bases	Mapped read	Mapped bases
NA12878 (CEU)	7	113.9M	33 bp	3.8G	34.9M	1.2G
NA18498 (YRI)	4	40M	35-46 bp	1.6G	28.2M	1.1G
NA18517 (YRI)	4	38.6M	35-46 bp	1.6G	28.1M	1.1G

Genotype calling accuracy was assessed using as gold standard Hapmap SNP genotype calls for the three individuals. To measure accuracy of genotype calling, we defined as true positive a correctly called heterozygous or homozygous non reference SNP and as false positive an incorrectly called homozygous SNP. We did not consider as error a heterozygous SNP called homozygous or not called because this can be due to lack of read coverage for one or both alleles. We consider that one method is more accurate than another when it is able to detect more true positives for the same number of false positives, or conversely if it detects the same number of true positives with fewer false positives. When assessing SNV detection accuracy, we define as true positive a detected heterozygous or homozygous non reference SNP, no matter which is the actual genotype call, and as false positive a homozygous reference SNP marked as having a variant. Thus, calling as heterozygous a homozygous not-reference SNP is considered a true positive for SNV detection, because the variant was detected, but a false positive for genotype calling because an inexistent reference allele is being called.

### Comparison of read mapping strategies

We used Bowtie [12] to map the reads against both the human reference genome (NCBI Build 37.1, downloaded from the UCSC hg19 genome browser database [25]) and the CCDS transcript library [19]. Table 3 gives read mapping statistics for the compared methods on the NA12878 dataset.

**Table 3:** Mapping statistics for the NA12878 dataset (million reads)

Run ID	Raw reads	Transcripts mapping	Genome mapping	Hard merge	Soft merge
SRR002052	12.6	2.9	4.3	4.5	4.7
SRR002054	12.9	3.9	5.7	5.9	6.2
SRR002060	25.7	4.4	6.7	7.0	7.3
SRR002055	11.4	3.7	5.5	5.6	5.9
SRR002063	23.0	3.5	5.6	5.8	6.0
SRR005091	13.9	3.3	4.9	5.0	5.2
SRR005096	14.4	0.6	1.0	1.1	1.1

Total	113.9	22.4	33.8	34.9	36.4

**Figure 1:**
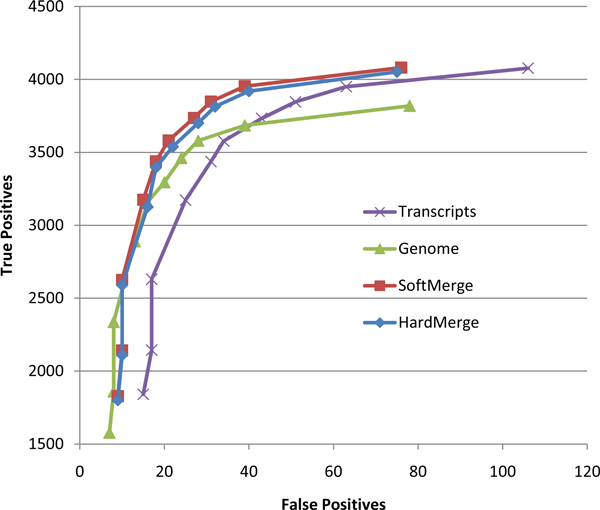
**Genotype calling accuracy for reads aligned uniquely to the reference genome, reads aligned uniquely to the CCDS transcripts, hard merged alignments, and soft merged alignments (NA12878 dataset using 41,961 Hapmap SNPs in CCDS exons as gold standard and SNVQ for genotype calling)**.

To assess the effect of various mapping strategies on genotyping accuracy, we ran SNVQ on datasets consisting of NA12878 reads mapped uniquely onto the CCDS transcript library and onto the reference genome, respectively reads mapped by the hard and soft merging strategies presented in the methods section. Since for reads mapped on transcripts it is only possible to detect SNVs in annotated exons included in the CCDS database, we excluded from this comparison all Hapmap SNPs located outside of annotated CCDS exons. Figure 1 shows that our merging strategies produce more accurate results than just genome or transcripts mapping for the NA12878 data. Although this comparison suggests that genome mapping could be more sensitive than the merging strategies for some specificity levels, we confirmed by repeating the comparison on the full set of Hapmap SNPs that merging methods dominate for all levels of specificity (data not shown). Since the performance of the hard and soft merging strategies is very similar, further results are presented only for the former method.

### Comparison of SNV calling and genotyping methods

**Figure 2:**
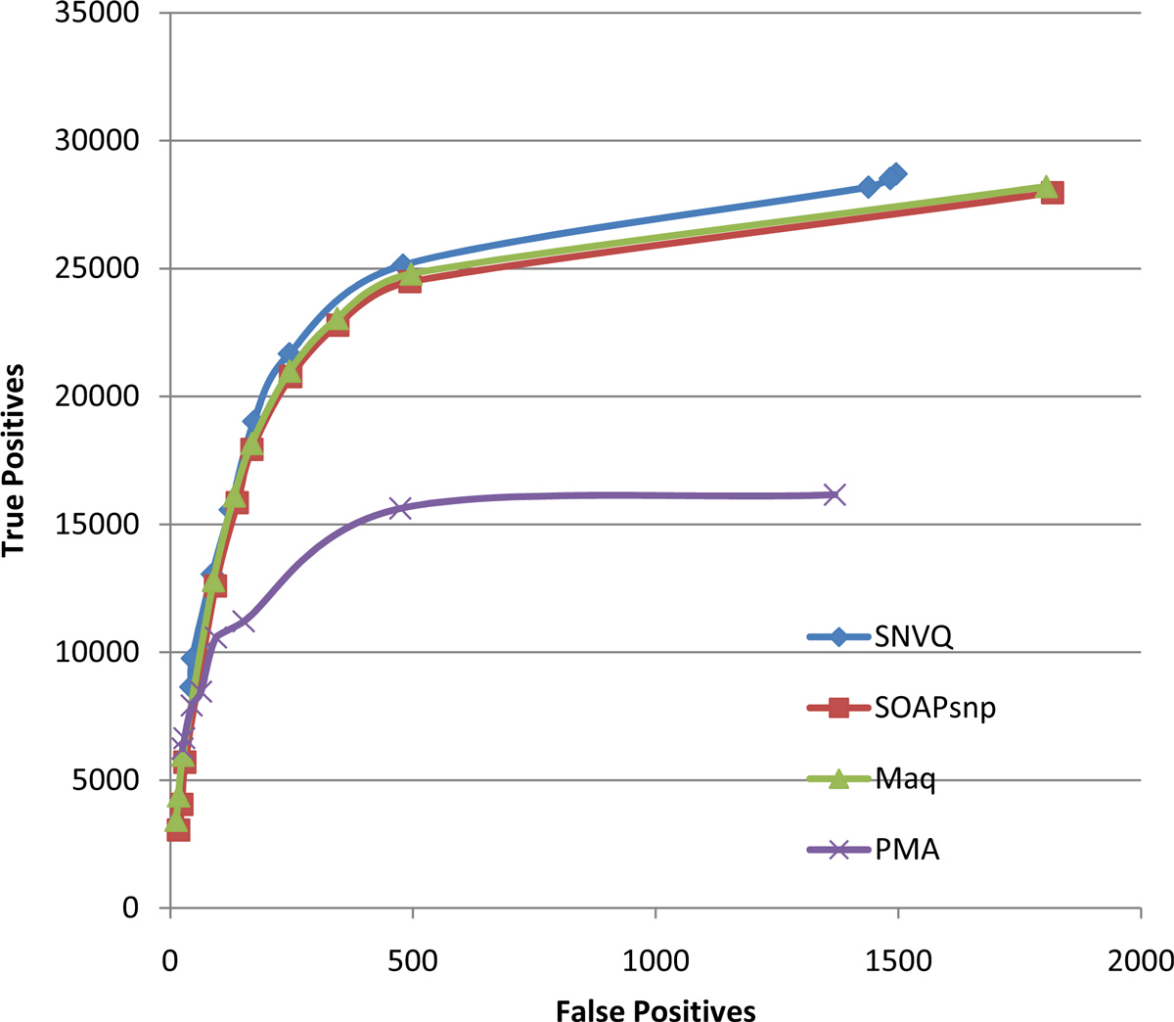
**Accuracy comparison for four different SNV detection methods on the NA12878 hard merged alignments**. The tradeoff between sensitivity and specificity is controlled in the Bayesian methods (SNVQ, SOAPsnp, and Maq) by varying the minimum probability of having a genotype different than the reference, while in PMA it is controlled by varying the maximum *p*-value required to reject the null hypothesis of absence of variants.

**Figure 3:**
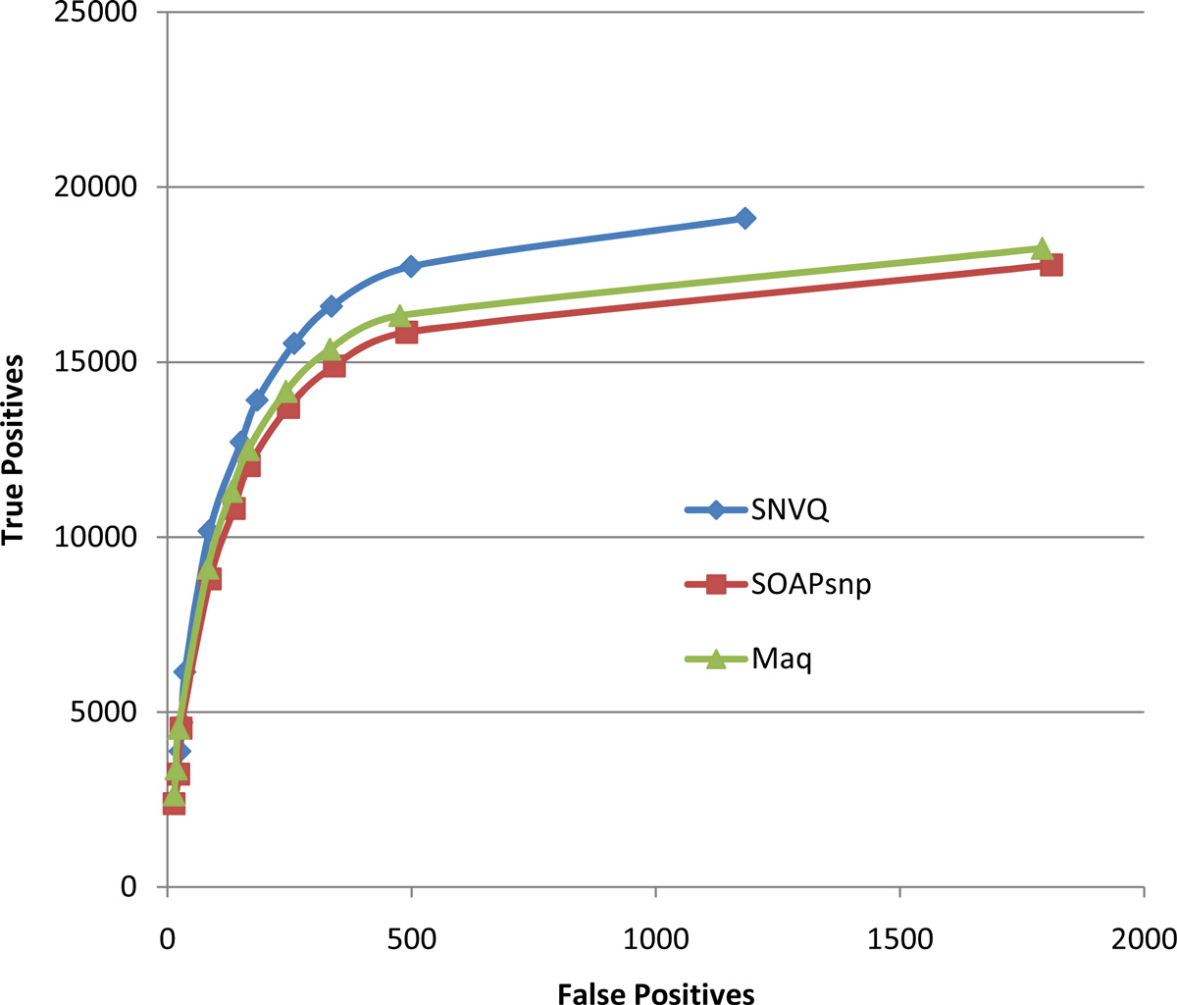
**Accuracy comparison among three different Bayesian methods for genotyping on the NA12878 hard merged alignments**.

We compared our SNVQ method with SOAPsnp [20] and Maq [11], two widely used Bayesian methods implemented in the SAMtools package [24]. We also experimented with the SNV detection method for mRNA reads of [26], called PMA, which is based in careful filtering of aligned reads and a binomial test equivalent to setting up a minimum coverage threshold to make a variant call relative to the total locus coverage. The trade-off between sensitivity and specificity of this method is controlled by the maximum *p*-value required to discard the null hypothesis of absence of a variant allele. In terms of outcome, both SOAPsnp and Maq have the a-priori advantage of not just pointing out the loci with variant alleles but also inferring the most likely genotype at each locus. The Bayesian methods also provide for each locus posterior probabilities of having an allele different than the reference and of the genotype itself. We ran all methods on the NA12878 reads aligned using the hard merge method. Figure 2 shows that all Bayesian methods have significantly better SNV detection accuracy than PMA and SNVQ is slightly more sensitive than SOAPsnp and Maq at different specificity levels obtained by varying the threshold on the genotype probability reported by each method. Figure 3 shows that the accuracy gain of SNVQ over SOAPsnp and Maq is more pronounced for genotyping accuracy. We confirmed this behavior by running the Bayesian methods on the set of reads mapped uniquely onto the genome reference (data not shown). Our results indicate that the binomial tests of heterozygosity employed by Maq and SOAPsnp result in under-calling true heterozygous loci. These heterozygous loci are found by SNVQ thanks to its unified model based on computing conditional probabilities for every possible genotype.

### Comparison of strategies for data curation

In practice SNV detection is the problem of separating allele calls that are different from the reference because of sequencing errors from calls that are different from the reference because they were sampled from a variant locus. With the current sequencing error rates, if sequencing errors were randomly distributed, it is not difficult to show that any of the discussed methods would have high accuracy. Unfortunately, each sequencing technology has different error patterns which can break the randomness assumption. In this section we describe three systematic error patterns characteristic to Illumina sequencing and assess how common ways to solve these issues performed in our testing data.

**Figure 4:**
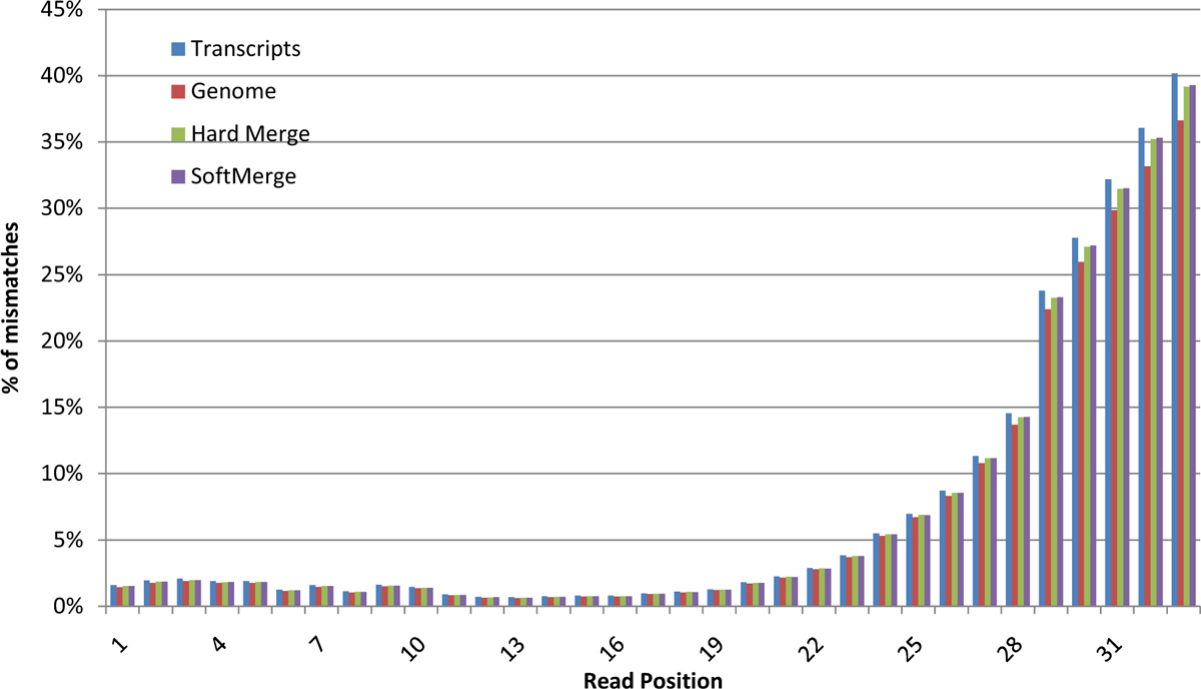
**Percentage of aligned reads with a mismatch with the reference genome per read position from 5' end to 3' end (NA12878 hard merged alignments)**.

**Figure 5:**
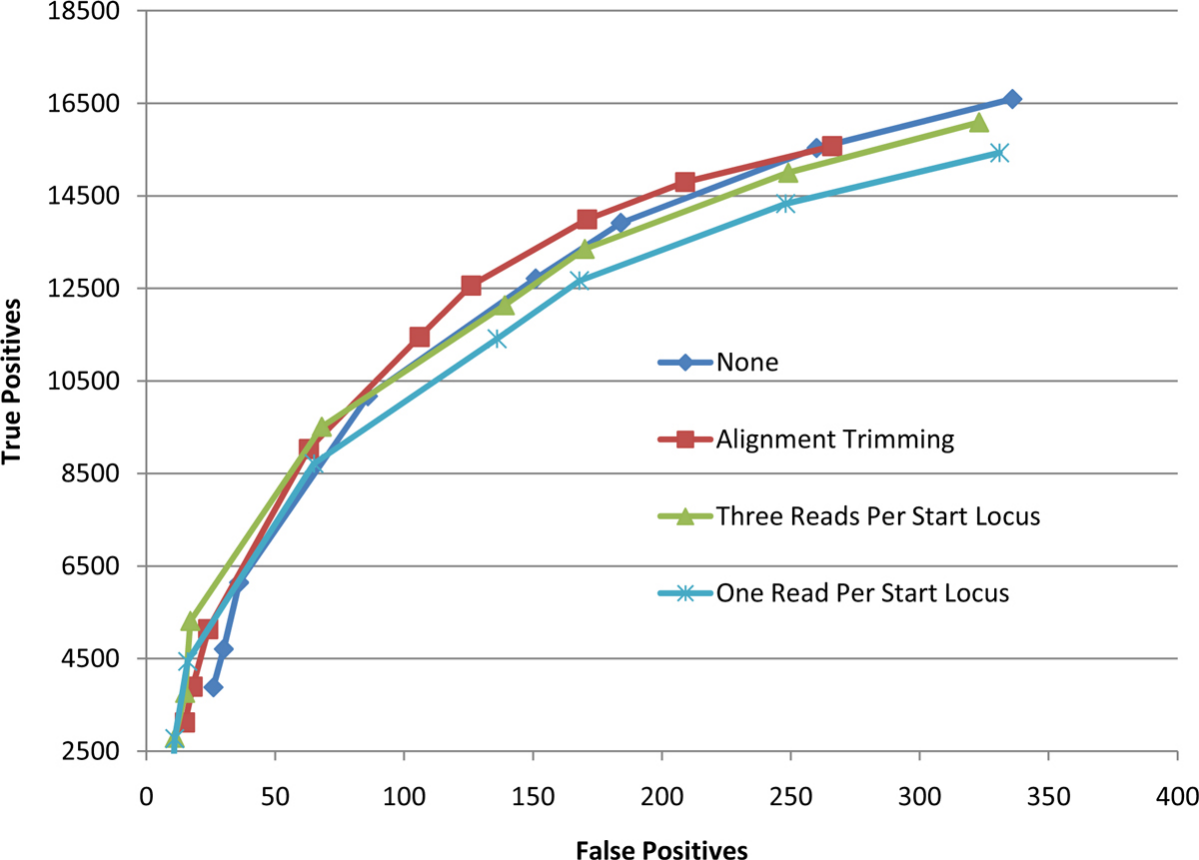
**SNVQ genotyping accuracy for three different filtering strategies applied to NA12878 hard merged alignments**. Results obtained without filtering are included for comparison.

A well-known error bias for Illumina reads (common in fact to all second-generation sequencing technologies) is that base calling errors tend to accumulate toward the 3' end of the reads due to a phenomenon referred to as *de-phasing *[27]. To test for this effect, we developed a module which calculates for a set of aligned reads the distribution of mismatches per read position from the 5' to the 3' end. In absence of any bias, this distribution should be close to uniform. As shown in Figure 4, the proportion of mismatches sharply increases towards the 3' end of the NA12878 reads. After observing this pattern in the mismatches rate, we decided to apply a filter on the aligned reads by disregarding the first base and the last 10 bases of each aligned read for SNV detection. Although this trimming strategy is equivalent to throwing away one third of the aligned bases for the NA12878 dataset, Figure 5 shows that this correction improves the specificity of the final calls without loosing sensitivity. Trimming aligned reads instead of raw reads is better because the bases sampled correctly in the trimmed region are still used to locate the correct location where the read must be aligned.

Another common source of false positive results are PCR amplification artifacts that produce a large number of copies of the same read [28]. One usual way to deal with this issue is to allow just one read to start at each possible locus. This filter eliminates artificial high coverage at every locus, which can be beneficial not only for discarding reads generated by PCR artifacts but also for normalizing biases produced by variable transcription levels. The main drawback of this strategy is that it can throw away useful read data, thus affecting sensitivity. An intermediate approach consists on allowing a small number *x *of different reads per start locus as described in [26]. Figure 5 shows that selecting just one read per start locus is indeed too restrictive for the NA12878 dataset but the three reads filter of [26] did not affect sensitivity and even improved it for stringent specificity requirements. Although the improvement is not as consistent as the one obtained by trimming aligned reads, we consider that this filter may be generally useful for correcting coverage biases without loosing sensitivity.

**Figure 6:**
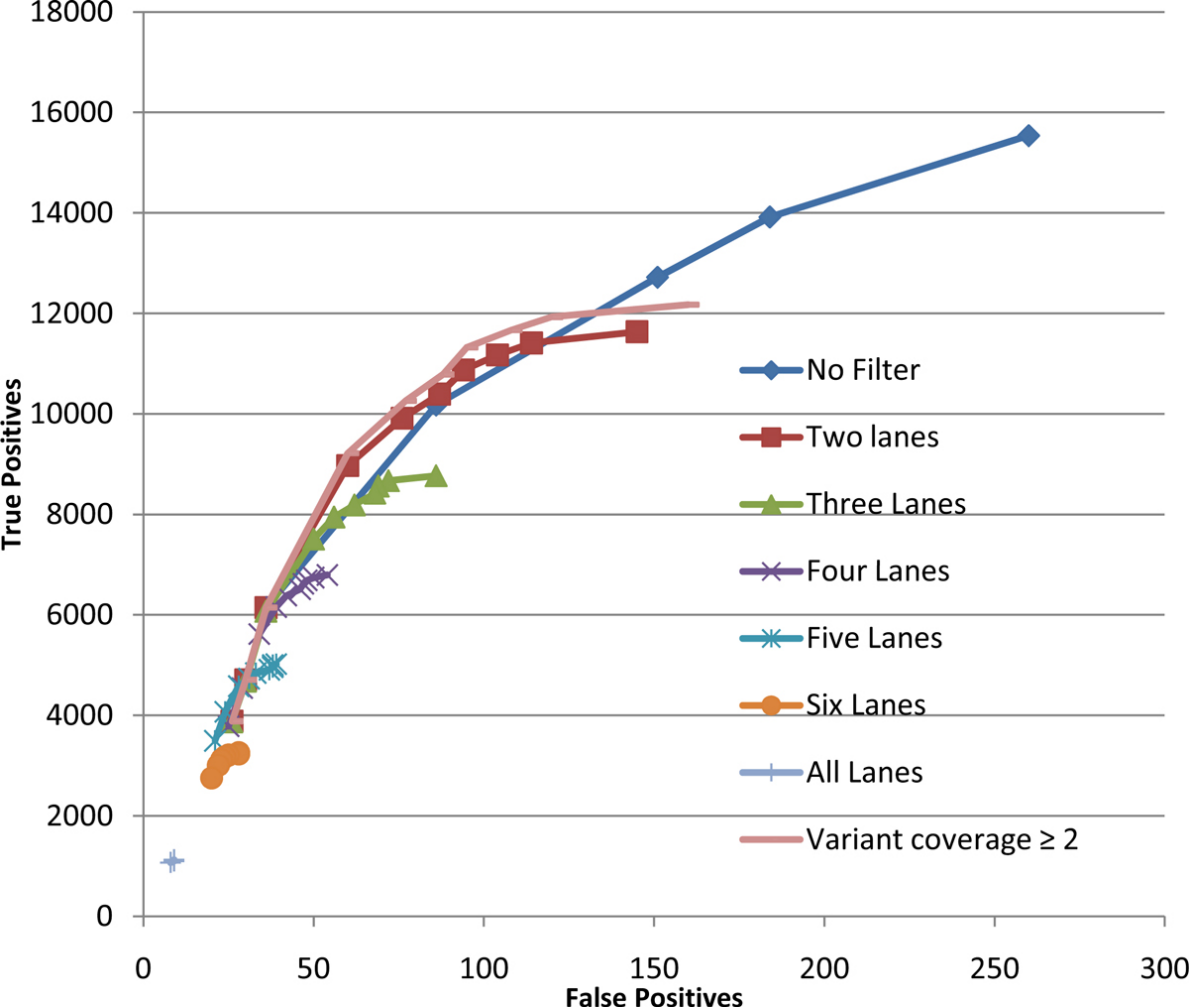
**SNVQ genotyping accuracy on NA12878 hard merged alignments for varying number of lanes where each alternative allele must be seen**. Results without filtering and with a threshold of at least two reads (regardless of their lanes of origin) are also included for comparison.

Finally, to control for the presence of correlated errors within individual lanes, a natural approach to increase specificity is to call a variant allele only if it seen in at least *x *different lanes, where *x *is a user specified parameter. We used NA12878 dataset, consisting of reads from seven different lanes, to assess the effect of the detection threshold on sensitivity and specificity. Figure 6 shows that after requiring a minimum of three lanes out of seven, the loss of sensitivity is larger than the improvement in specificity. We compared also the simple rule of keeping variants passing the threshold of observing at least two times the non reference allele with the more stringent rule of observing the non reference allele in at least two different lanes. We found that the first filter produced slightly better accuracy for this dataset.

### Effect of coverage on genotyping accuracy

**Figure 7:**
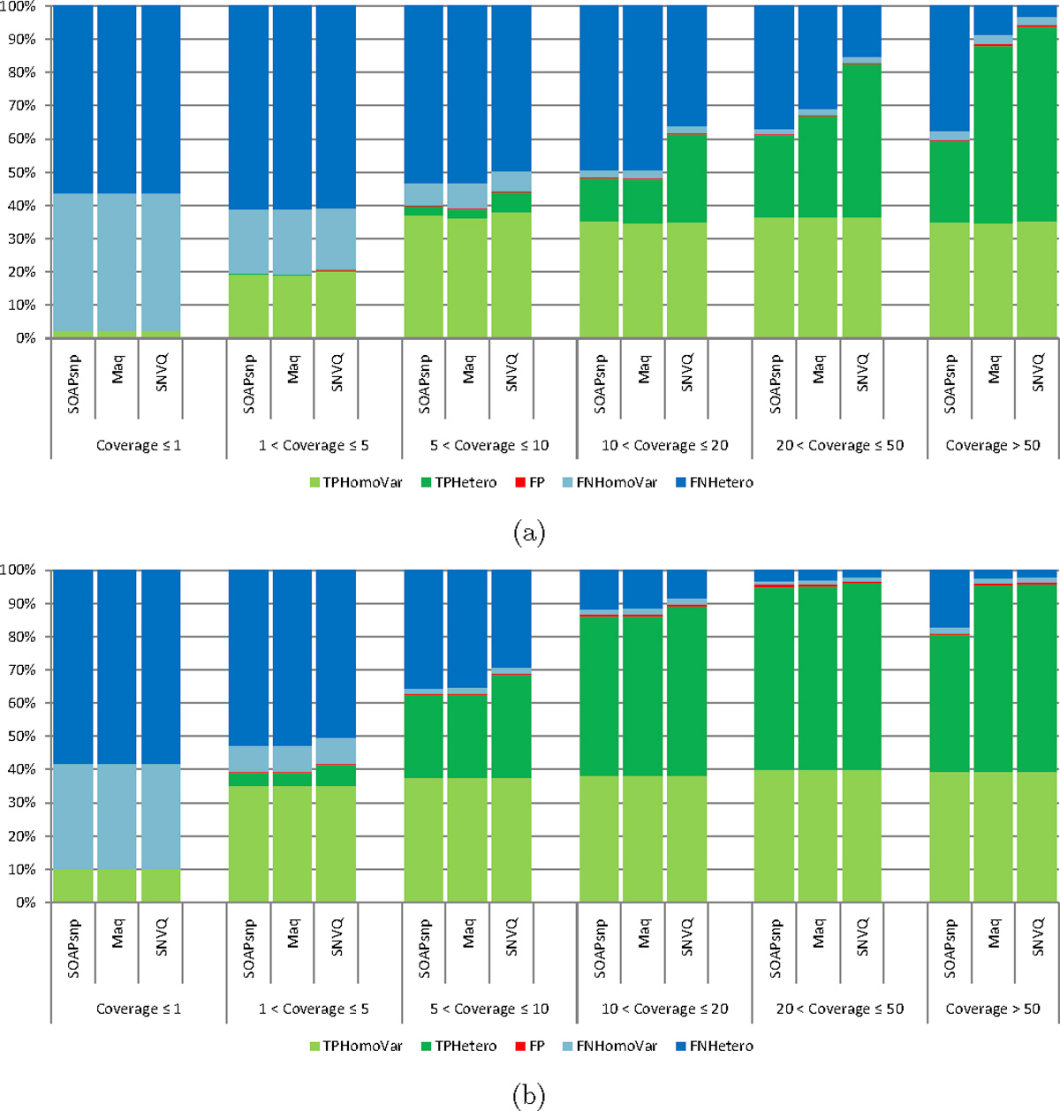
**Percentage of SNVQ true positive, false positive, and false negative alleles as a function of average exon coverage for NA12878 (a) and NA18517 (b) datasets**.

RNA-Seq reads are sampled from transcripts roughly proportionally to their relative expression levels, resulting in uneven coverage of different variants. To assess the effect of this uneven coverage on genotyping accuracy we calculated the average coverage for every exon in the CCDS database based on the hard merged alignments. Average exon coverages are proportional to the RPKM (Reads per Kilobase per Million Reads) values more commonly used to report expression levels for RNA-Seq data, and indeed can be inferred from RPKM values by taking into account exon lengths and the total number of mapped reads. Figure 7 shows genotyping accuracy achieved by SOAPsnp, Maq, and SNVQ on the NA12878 and NA18517 datasets, computed for several bins of variants grouped according to the average coverage of the exon to which they belong. As expected, all methods have poor sensitivity for variants with low coverage. Owing to improvements in sequencing data quality, all methods have improved genotyping accuracy on the more recent NA18517 data compared to NA12878. SNVQ consistently outperforms the other two methods, with most pronounced gains at intermediate coverage depths and on the lower quality NA12878 data.

### Genotype calling from heterogeneous samples

**Figure 8:**
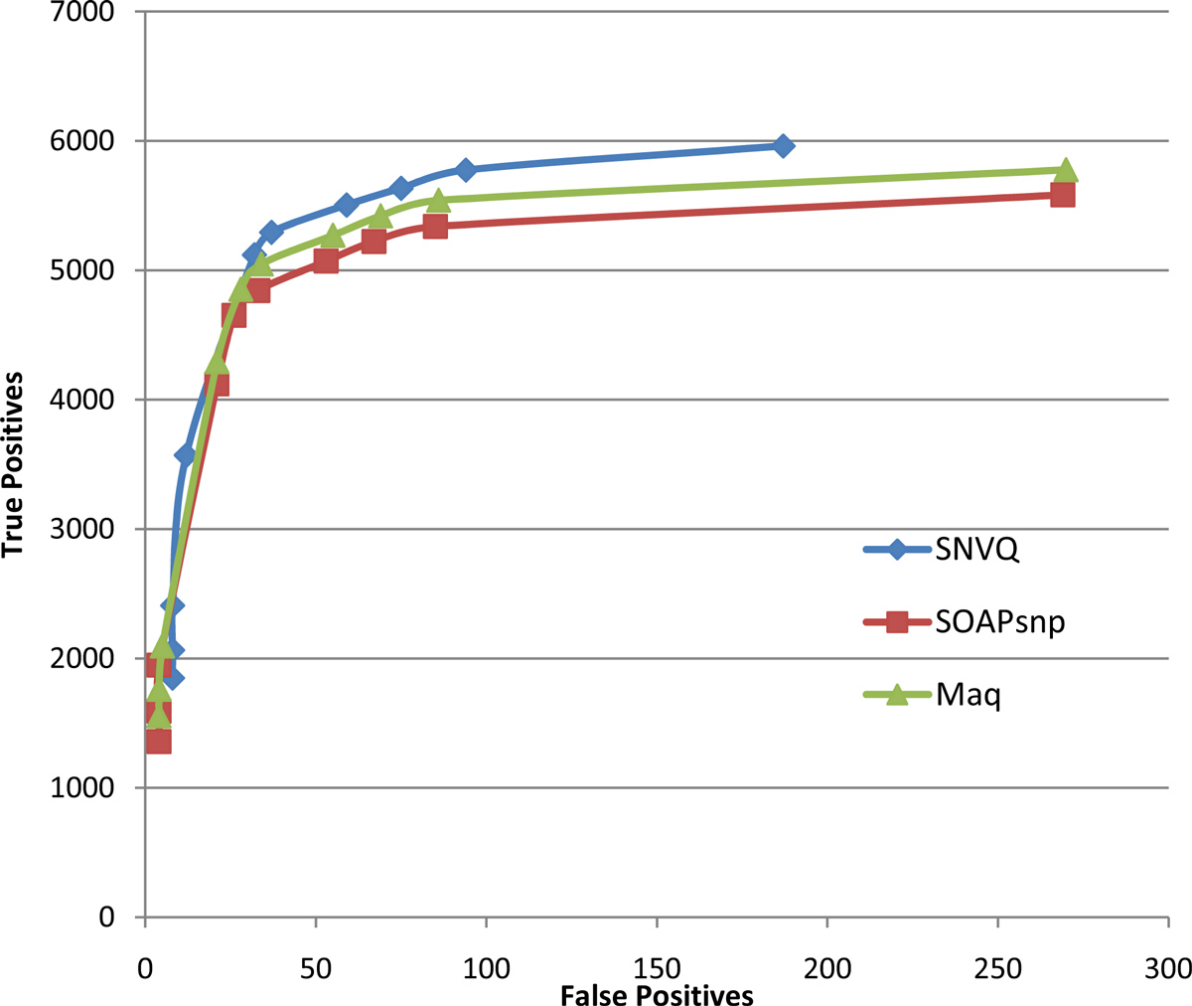
**Genotyping accuracy of SNVQ, SOAPsnp, and Maq on RNA-Seq reads pooled from Hapmap individuals NA12878, NA18498, and NA18517**.

**Figure 9:**
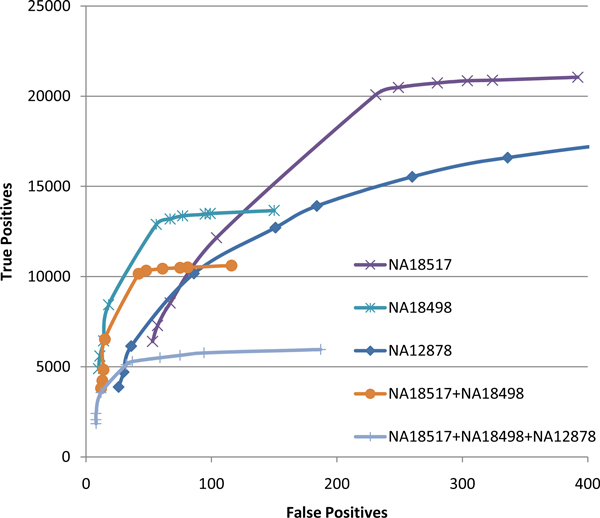
**SNVQ genotyping accuracy on individual and pooled RNA-Seq reads from Hapmap individuals NA12878, NA18498, and NA18517**.

A main motivation for this work has been identification of expressed non-synonymous somatic mutations in cancer tumors, a subset of which are predicted to yield tumor-specific epitopes with significant immunotherapeutic potential [29]. In addition to uneven expression levels, variant calling from cancer RNA-Seq data is further complicated by the typically heterogeneous nature of these samples. In order to assess the effect of such heterogeneity on genotyping accuracy in a context in which the true genotypes are well characterized we performed experiments on pooled RNA-Seq reads from the three Hapmap individuals. As shown in Figure 8, the relative performance of the three compared genotyping methods on the pooled data is similar to that observed for individual samples (compare, e.g., to Figure 3); with SNVQ having a small advantage over SOAPsnp and Maq. However, at a fixed specificity level, the sensitivity achieved by all methods is significantly reduced on the pooled sample. This is illustrated in Figure 9, where tradeoff accuracy curves are plotted for SNVQ calls made from individual RNA-Seq datasets as well as RNA-Seq reads pooled from the two Yorubans, and all three Hapmap individuals, respectively. While the accuracy drops with increased heterogeneity, SNVQ retains the ability to call a significant fraction of variants with high specificity.

## Conclusions

Second generation sequencing of mRNA is becoming the method of choice to investigate the behavior of human cells and to reveal the functional effects of variation. In this paper we introduced a mapping strategy for mRNA reads which fully utilizes the information contained in both the reference genome sequence and reference databases of known transcripts such as CCDS. We also presented a Bayesian model for SNV detection and genotyping called SNVQ that seeks to improve genotype calls by fully exploiting the information contained in base quality scores. Finally, we performed a comparison among commonly used mapping, SNV detection and genotyping methods, and data curation strategies with the aim to select the most effective methods to identify expressed single nucleotide variants from RNA-Seq data, taking advantage of the availability of RNA-Seq data for Hapmap individuals with well characterized genotypes. Our experiments indicate that the double reference mapping and merging strategy yields improved SNV calling and genotyping accuracy compared with methods based on mapping to a single reference. The experiments further suggest that SNVQ achieves improved accuracy over existing methods, and retains its power to detect variants with a high specificity even from heterogeneous RNA-Seq samples.

In future work we seek to integrate our tools with methods for estimating isoform expression levels [30] and to extend our model by incorporating allele specific expression of isoforms [8]. We also plan to integrate additional transcript annotation sources such as dbEST and UCSC, and to integrate our methods in a bioinformatics pipeline enabling personalized cancer immunotherapy based on tumor transcriptome sequencing.

## Competing interests

The authors declare that they have no competing interests.

## Authors' contributions

PKS and IIM conceived and supervised the study. JD designed and implemented the algorithms, conducted the experiments, and drafted the manuscript along with IIM. All authors have read and approved the final manuscript.
